# Vitamin B12 Deficiency Presenting as Psychotic Symptoms in a Psychiatry Department: A Case Report

**DOI:** 10.7759/cureus.50492

**Published:** 2023-12-14

**Authors:** Xiabing Zheng, Riyang Qiu, Wenya Zhang, Xiaohong Chen, Man Wang

**Affiliations:** 1 Department of Precision Therapy, Shenzhen Kangning Hospital, Shenzhen, CHN; 2 Department of Psychiatry, Shenzhen People’s Hospital, Shenzhen, CHN

**Keywords:** case report, unbalanced diet, psychotic symptoms, anemia, vitamin b12 deficiency

## Abstract

Vitamin B12 deficiency is a metabolic disorder affecting the functions of hematopoietic system and nervous system. It manifests as a wide spectrum of anemia and nervous system symptoms, but psychotic symptoms are rare. We report a case of a 34-year-old man with an unbalanced diet who initially presented with psychotic symptoms. Prompt remission was achieved through vitamin B12 repletion, ultimately leading to a diagnosis of mental disorder caused by vitamin B12 deficiency. The patient provided his written informed consent to participate in this study. This case report emphasizes the importance of ruling out vitamin B12 deficiency in patients with psychotic symptoms instead of directly diagnosing schizophrenia, especially in cases where diet is unbalanced.

## Introduction

Vitamin B12 is a water-soluble vitamin that is crucial for neurologic function and the hemopoietic system [1]. Vitamin B12 is mainly obtained from meat products, dairy products, fortified cereals and other supplements, and is absorbed at the end of the ileum with the help of intrinsic factor produced by the stomach’s parietal cells [2]. The causes of vitamin B12 deficiency mainly focus on pernicious anemia, insufficient dietary intake, and malabsorption.

The clinical manifestations of vitamin B12 deficiency can affect multiple systems, and the severity of symptoms can vary between individuals. Among the clinical symptoms caused by the lack of vitamin B12, anemia and nervous system are the most common [2]. The patient may have anemia manifestations such as fatigue, lethargy, paleness and dry lips, and taste disorders, as well as neurological manifestations, including myelopathy, neuropathy, dementia, and rarely cerebellar ataxia and dyskinesia.

However, only a few studies mentioned the emergence of mental symptoms [3,4]. Further understanding of the clinical manifestations of vitamin B12 deficiency will help clinicians in making early diagnoses and timely treatment decisions, thereby preventing unnecessary treatments, hospital admissions and serious complications. In this study, we present a case of a 34-year-old patient whose features were initially suggestive of schizophrenia but was later diagnosed with vitamin B12 deficiency. Through this case, we gained a deeper understanding of vitamin B12 deficiency and were able to provide timely and accurate intervention and treatment measures.

## Case presentation

The patient was a 34-year-old male who was forcibly admitted to the emergency department of a psychiatric hospital with the assistance of his family. The patient began to exhibit depression syndrome half a year before admission, including depressive mood, decreased interest, retardation of thinking and other symptoms. Based on these symptoms, the patient was diagnosed with major depressive disorder, which persisted despite antidepressant treatment. The patient began to show psychotic symptoms one week ago, including verbal hallucination, persecutory delusion, relational delusion, evil delusion, a sense of being monitored, and repeated suicide attempts, which were not systematic and fixed, and different delusional content was obtained after repeated questioning. There were no urinary symptoms, limb numbness, gait instability, nausea and vomiting or other symptoms in this patient. There was no history of major somatic diseases, such as gastrointestinal problems. The patient had an unbalanced diet, avoiding red meat like pork or beef since childhood but consuming fish once a week. He had a history of poor academic performance, consistently ranking last in his class, and had repeated a grade before eventually dropping out of junior high school in the second year. Besides, the patient had a history of multiple jobs and two unsuccessful blind dates, but remained unmarried. He was not fond of tobacco or wine.

On physical examination, his vital signs were stable. Height was 173 cm, weight was 55 kg and body mass index (BMI) was 18.38. He had anemic appearance, pale complexion, sclerae and skin mildly icteric, and dry hair. Cardiopulmonary and abdominal examination was normal. During the examination of the neurological system, the patient's pupil size and reflex were found to be within normal limits. The cranial nerve examination revealed no abnormalities. However, the patient was unable to fully cooperate with the sensory and motor examinations. The tendon reflex of the extremities was noted to be hyperactive, while there were no signs of pathological or meningeal stimulation.

**Figure 1:**
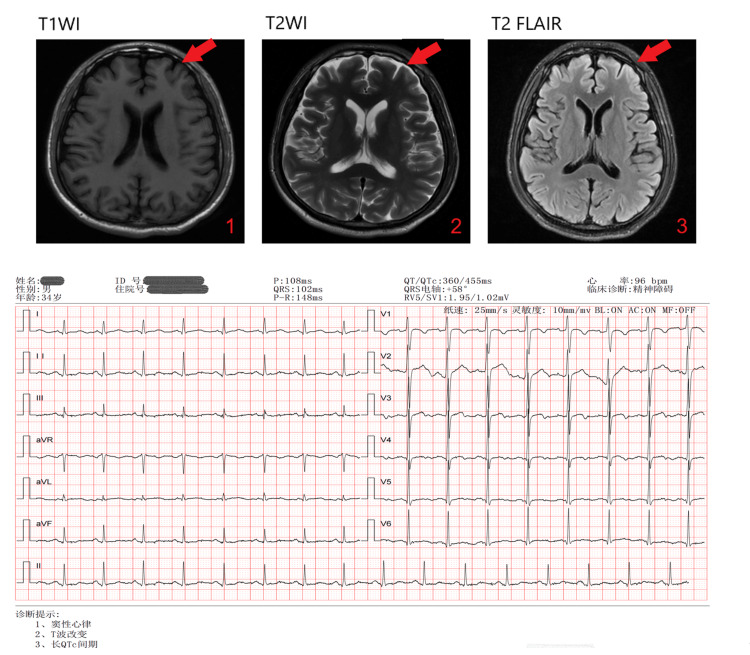
The ECG and MRI results at admission. ECG: electrocardiogram, MRI: magnetic resonance imaging, FLAIR: fluid-attenuated inversion recovery

The initial blood tests showed erythrocytopenia with severe macrocytic anemia, elevated lactate dehydrogenase (LDH) and homocysteine, with low serum vitamin B12, as seen in Table 1. No abnormality was found in genetic and metabolic genome detection. Infectious disease test was negative. Electrocardiogram (ECG) examination showed: 1. sinus rhythm; 2. T wave changes; 3. long QTc interval. Magnetic resonance imaging (MRI) of the head suggested that bilateral frontal sulci were widened. The ECG and MRI are shown in Figure 1. After three days of treatment, the levels of methylmalonic acid in serum and urine were supplemented and checked, and the results were normal. Intrinsic factor and gastric parietal cell antibody tests were conducted at a comprehensive hospital several weeks after the patient was discharged, and the results were normal.

**Table 1:** Levels of blood parameters before and after treatment. RBC: red blood cells, MCV: mean corpuscular volume, MCH: mean corpuscular hemoglobin, MCHC: mean corpuscular hemoglobin concentration

Laboratory test	At admission	Two weeks after treatment	One month after treatment	Reference ranges
RBC count (million/mcl)	2.1	3.1	4.72	4.3-5.8
Hemoglobin (g/dl)	8.2	10.8	14.4	13.0-17.5
MCV (fL)	106.6	105.1	92.6	82.0-100.0
MCH (pg)	38.7	34.7	30.5	27.0-34.0
MCHC (g/L)	363	330	330	316-354
Total bilirubin (μmol/L)	49.3	13.1	14.6	<23.0
Direct bilirubin (μmol/L)	16.7	2.3	2.5	<8.0
Indirect bilirubin (μmol/L)	32.6	10.8	12.1	<20.0
Lactate dehydrogenase (U/L)	1173	454	140	120-250
Homocysteine (μmol/L)	16.0	7.3	7.2	<15
Serum VB12 (pmol/L)	10	>1144	1088	133-675
Serum folic acid (nmol/L)	45.9	>51.7	>51.7	7.0-45.2

Considering the prominent psychiatric symptoms of the patient, the admission diagnosis was considered schizophrenia, but the antipsychotic treatment did not yield satisfactory results. Accordingly, considering the patient's fluctuating mental symptoms, abnormal hematological test results and dietary habits, it tended to be diagnosed as a mental disorder caused by vitamin B12 deficiency. Based on this diagnosis and according to treatment guidelines, the patient received intramuscular injections of vitamin B12 at 1 mg per day for one month, followed by oral vitamin B12 supplementation with concurrent low-dose aripiprazole treatment. After receiving four weeks of treatment, the psychotic symptoms and anemia manifestations of the patient were significantly improved, and the patient could clearly recall the whole course of the disease and adhere to the entire treatment process.

It was interesting to note that the patient had hypomania after one week of treatment, including elevated mood, excessive speech, increased appetite, which was incongruous with the patient's prior personality. The manic manifestation began to subside after half a month of treatment, and as the manic symptoms subsided. Levels of blood parameters before and after treatment are shown in Table 1.

## Discussion

Vitamin B12 plays an important role in erythropoiesis, maintaining the structure of nerve myelin sheath, and DNA synthesis. As a water-soluble vitamin, the recommended intake of vitamin B12 is 3 ug/day. It cannot be synthesized in the human body and the only natural food source is animal products [5]. Symptoms caused by vitamin B12 deficiency often focus on erythropoiesis, nerve damage, cognitive function and other fields. Vitamin B12 deficiency is common, with important clinical consequences and variable clinical manifestations, especially in the general economic conditions and vegetarian groups. When vitamin B12 is deficient, it will affect the human body through three pathways, including the conversion of methyl alcohol acid to successful coenzyme A; the conversion of homocysteine to methionine; and the conversion of 5-methyltetrahydrofolate to tetrahydrofolate.

The causes of vitamin B12 deficiency are various, mainly due to food-bound cobalamin malabsorption [6]. Vitamin B12 deficiency often occurs in pregnant women, infants, children and other groups, especially in vegan or vegetarian communities. The content of vitamin B12 in the body is about 1-5 mg, while the daily consumption is 2-3 μg, in which about 50-60% is absorbed [7,8]. The patient in this report was gradually lacking vitamin B12 due to the unbalanced diet. However, with the vitamin B12 hepatointestinal pathway for bacterial B12 absorption, it takes 10-20 years for vegetarians to show the symptoms of vitamin B12 deficiency [1]. At the same time, although fish was not as good as meat in providing vitamin B12 [5], it still delayed the time for the patient to seek medical treatment and he did not show mental symptoms requiring treatment until the age of 34.

As a common disease, vitamin B12 deficiency can manifest clinically as non-specific symptoms and lacks specificity in diagnosis [6]. Typical clinical manifestation of vitamin B12 deficiency is anemia, including weakness, palpitation, fatigue, pale complexion, dizziness and shortness of breath, and jaundice caused by hemolysis [1]. This case is notable for the patient's initial presentation to a psychiatric hospital with fragmented and unsystematic psychotic symptoms that differed from those typically associated with schizophrenia. In our daily ward rounds, patients often tell different delusions content. The patient exhibited depressive symptoms, including unexplained self-harming behavior, repeatedly hitting his head against surrounding objects, which could not be attributed to psychotic symptoms. Despite treatment, the patient remained unable to provide a reason for his head-hitting behavior. It is the ambiguity of symptoms that prompts us to further rule out the possibility of organic diseases. Previous studies have also illustrated that vitamin B12 deficiency can lead to psychiatric symptoms [4,9].

Different from the long treatment time required for neurological symptoms due to the vitamin B12 deficiency [10], it was observed that the patient’s symptoms were greatly relieved in a relatively short time in this case. Especially, a hypomanic state was observed for several days after one week of intramuscular injection of vitamin B12. The mechanism by which vitamin B12 deficiency leads to the onset of symptoms was unclear and could be related to the failure of methylation reactions [11], but treatment outcomes suggested that timely vitamin supplementation was proven to be beneficial to the disease. Therapeutic trials also verified the important impact of vitamin B12 deficiency, and the increase in red blood cell count and hemoglobin concentration during hospitalization was consistent with the improvement of the patient's condition.

A vitamin B12 level below 200 ng/L is sensitive enough for making a diagnosis of vitamin B12 deficiency [12]. The long-term dietary habits, physical examination, serum vitamin B12 level, and temporal correlation of the outcome of psychotic symptoms after vitamin B12 supplementation helped correlate the patient's condition with vitamin B12 deficiency. Studies believe that serum holocobalamin, blood homocysteine and methylmalonic acid can become better reflections of the vitamin B12 status in the body [1], but due to the limitations of equipment in psychiatric hospitals, these tests have not been improved in time. Although methylmalonic acid was detected on the third day after vitamin treatment, considering that the metabolic process usually restores excretion to normal in a few days of vitamin B12 treatment [1], it was difficult to decide its significance.

## Conclusions

This case report describes a patient with an unbalanced diet who initially presented with psychiatric symptoms due to vitamin B12 deficiency and was subsequently admitted to a mental health facility. The study highlights the importance for clinicians to remain vigilant for vitamin B12 deficiency in patients with psychiatric symptoms, particularly when encountering fragmented and unsystematic psychotic symptoms along with dietary imbalances.
